# Increase in forest structural complexity along a precipitation gradient is mediated by partial harvests in temperate Patagonian forests

**DOI:** 10.1038/s41598-024-64523-5

**Published:** 2024-06-13

**Authors:** Daniel P. Soto, Dominik Seidel, Ángela Hernández-Moreno, Klaus J. Puettmann, Pablo J. Donoso

**Affiliations:** 1 Departamento de Ciencias Naturales y Tecnología, Universidad de Aysén, Coyhaique, Chile; 2 Department for Spatial Structures and Digitalization of Forests, Georg-August-Universität, Göttingen, Germany; 3 Centro de Investigación en Ecosistemas de la Patagonia (CIEP), Coyhaique, Chile; 4 Department of Forest Ecosystems and Society, Oregon State University, Corvallis, OR USA; 5 Instituto de Bosques y Sociedad, Universidad Austral de Chile, Valdivia, Chile; 6 Departamento de Ecosistemas y Medio Ambiente, Facultad de Agronomía y Sistemas Naturales, Pontificia Universidad Católica de Chile, Santiago, Chile

**Keywords:** Forestry, Forest ecology

## Abstract

Increasing forest structural complexity is becoming a common goal in forestry worldwide. However, the lack of empirical quantification clouds its implementation. Here we quantified the long-term effects (> 30 y) of partial harvest on stand structural complexity and net primary productivity using the east–west precipitation gradient (318–2508 mm, mean annual precipitation-MAP) of western Patagonian as a study system. In this gradient, pairs of 1-ha plots on 20 sites (20 plots harvested and 20 plots unharvested) were installed. In each plot terrestrial laser scanning was used to quantify the stand structural complexity index (SSCI), and Sentinel satellite images to obtain the Enhanced Vegetation Index (EVI: proxy of net primary productivity). Generalized linear mixed-effect models were used to relate SSCI to MAP and EVI to SSCI, with harvesting as indicator variable, and site as random variable (two plots nested to same precipitation). Results showed that harvested plots on mesic-to-humid sites (but not on dry sites) had higher SSCI and EVI values compared to unharvested plots, likely due to a greater vertical canopy packing. These results show the influence of precipitation on SSCI, which resulted in a more diversified stand structure and higher EVI. Such insights support site-specific management aimed to increase forest structural complexity.

## Introduction

Forestry in the XXI century deals with challenges that derive from increased amount and variety of societal demands from forests, including the provision of wood, conservation of biodiversity, ensuring sufficient amounts of high quality and quantity of water, and carbon sequestration to mitigate climate change^1–6^. In contrast to the main emphasis on wood production in the past, which has resulted in efforts to homogenize stand forest structures, the emphasis on multiple ecosystem services has increased interest in forests with higher compositional diversity and structural complexity^3–5,7^. Structural complexity is reflected in variable vertical and horizontal structures (e.g., tree sizes, tree heights, spatial heterogeneity)^8–10^, dead wood (snags and downed wood), rich understories^4,11^ and fungal diversity^12^. Recent conceptual advances suggest that such forests are also better able to adapt to a variety of present and future and novel environmental conditions and disturbances^3,5,13^. However, recent advances in scanning technology, specifically ground based LiDAR provide for efficient data collection and quantification of stand structure^14,15^.

The concept of forest structural complexity has spurred a productive research area^15^. Efforts began to quantify the distribution of trees and their canopies in three-dimensional space, and to relate this quantification to “standard” forest attributes such as biomass, basal area, volume, leaf area or canopy height^16–21^. With detailed measurements, greater forest structural complexity could be related to higher diversity of tree sizes and crown morphologies^17,22,23^. Also, greater structural complexity relates to more densely packed forest canopies, and to a high degree of heterogeneity of tree-sizes and biomass distribution in the three-dimensional space^17,24^. Measuring forest structural complexity has shown the potential to increase the understanding of the relationships of three-dimensional forest structure and ecosystem processes and functions^15,17,25^. However, few empirical studies analyze drivers of variation in forest structural complexity, e.g., rainfall and ecosystem processes, respectively^17,23^, and even less studies refer to silvicultural implications on structural complexity.

Recent advances in airborne and terrestrial LiDAR (Light detection and ranging) technologies allow quantification of the three-dimensional nature of the forest structure “holistically” (sensu^15,26,27^) or in a “non-feature-centered” manner (sensu^28^). Such measures of forest structural complexity, e.g. the stand structural complexity index-SSCI^16^, the box-dimension-Bd^15^ or canopy rugosity^29^, operate without considering individual attributes, such as tree density, basal area, diameter at breast height, leaf area, volume and/or biomass, but instead attempt to characterize all above ground forest elements together^17,26^. For example, the box-dimension approach quantifies structural complexity from 3D forest models obtained from LiDAR scans that include all elements in a forest scene without a need for segmentation of the data^30^. Such measures triggered the development of new methodologies and metrics to quantify forest structural complexity^16,27,31^. Because anthropogenic and natural disturbances influence forest structure, holistic measures of forest structural complexity have been increasingly used to study the impacts of forest management and forest disturbances on biodiversity and productivity^17,22,29,32,33^. For example, holistic measures of forest structural complexity using terrestrial laser scanning (TLS) were positively related with net primary productivity^22,32,34^. Other studies evaluated the relationships of forest structural complexity with forest management^16,35,36^, tree mixtures^33,37^, plant species diversity^17,33^ and forest microclimate^38^. Zemp et al.^39,40^ also studied how restoration plantings with native tree species enhanced forest structural complexity and improved biodiversity in oil palm monocultures. All these recent examples indicate the great potential for using TLS to assess forest structural complexity and its relationship with forest ecological attributes and forest management practices. However, all these studies that documented short- and mid-term effects of treatments provide only limited understanding of the impacts on slower processes, such as succession, development of structural complexity and associated changes in growing^41^ conditions and patterns^42^.

In western Patagonia, partial or low intensity harvesting operations have been widely practiced since the first wave of immigrants that came to the Aysén region of Chile^43^. Many partially harvested forests have been left relatively untouched. In our study sites, harvested stands have not had any further potential treatments since the 1970–1990s (Daniel Soto, personal observation). Thus, only natural forest reorganization and succession have shaped stand development and environmental conditions after the harvests^44^. A recent study showed that the region is relatively stable in terms of land use changes and anthropogenic influences over the last decades^45^. Therefore, the region contains forests that were partially harvested over 30 years ago intermixed with unharvested forests^46^. Having forests with different stand histories in close vicinity, and a wide range of climate conditions (e.g., mean annual precipitation ranging from 2508 mm in the west to 318 mm in the east within ~ 200 km distance) represents an exceptional opportunity to test the hypothesis of whether precipitation drives forest structural complexity in previously harvested and untreated forests using the well-established and validated LiDAR-based stand structural complexity index (SSCI) developed by Ehbrecht et al.^16^. Novel insights by comparing long-term harvested and untreated forest conditions not only can provide guidelines for forest managers interested in increasing structural complexity, but also will complement the global assessment of forest structural complexity recently conducted by Ehbrecht et al.^17^. These authors used a similar precipitation gradient (500–2500 mm) to ours, but selected sites at a global, and not regional scale. The environmental setting of the Aysén region in western Patagonia represents an opportunity to test the following hypotheses.

**H1:** The SSCI (stand structural complexity index) is positively related to MAP and this relationship is influenced by stand disturbance history. We hypothesize that past harvesting activities increase the strength of the precipitation-structure relationship towards humid sites, which have fewer environmental limitations compared to dry sites (drier and windy conditions at the ecotone with steppe), showing a climate-disturbance history interaction, i.e., with precipitation.

**H2:** Net primary productivity (i.e., EVI) is positively related to SSCI and this relationship is influenced by stand disturbance history. We hypothesize that net primary productivity at sites with high SSCI will be higher due to the development of new cohorts in canopy gaps created following harvestings, and lower at sites with low SSCI with environmental constrains that limit tree regeneration in gaps following disturbances.

**H3:** A positive triple interaction between SSCI, MAP and disturbances (harvesting) improves the EVI, in mesic and humid sites through relatively quick tree regeneration after harvesting. In contrast, EVI is lower after harvesting on dry sites due lack of regeneration, even after longer periods.

## Material and methods

### Study area

**Figure 1:**
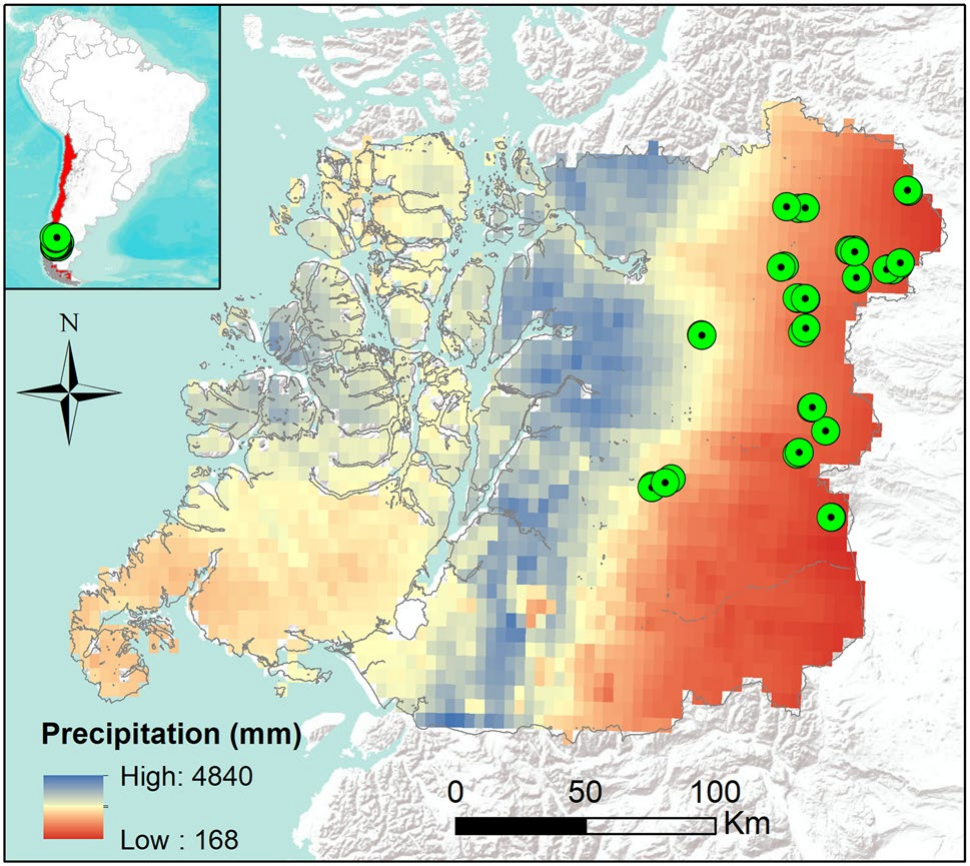
Study area and precipitation sites displayed throughout the Aysén region in western Patagonia. The map was made using ArcGIS 10.8.1 software (ESRI, Redlands, Ca.) with the integration of the precipitation raster obtained from CR2MET (www.cr2.cl/datos-productos-grillados) of the Center for Climate and Resilience Research (www.cr2.cl). Green dots show location of studied plots.

We selected 20 sites with pure lenga (*Nothofagus pumilio*), and mixed lenga-coihue (*Nothofagus dombeyi*) old-growth forests in the Aysén administrative region of Chile, also known as Western Patagonia were selected across a precipitation gradient from 318.3 mm MAP (mean annual precipitation) to 2508.2 mm of MAP (Fig. 1). Specifically, 15 sites were pure lenga-dominated old-growth forests, which span from the ecotone with steppe with 308.3 mm of MAP in Parque Nacional Cerro Castillo (Ibañez sector) to 1582, 1 mm of MAP in Reserva Mañihuales, and 5 sites mixed with lenga-coihue dominated old-growth forests were selected from mesic (Reserva Nacional Coyhaique (1109.6 mm MAP) and moist (Valle Laguna: 2508,2 mm MAP) conditions (Table 1). For mixed sites in general coihue has larger diameter than lenga (Dg), and the proportion of lenga basal area (pBA) was decreasing when precipitation increases in both conditions, unharvested and harvested (Table 1). These sites were selected across places that had past harvesting activities following the Chilean colonization in the region during early twentieth century, which corresponded to low-intensity partial overstory harvests conducted above 30 years ago. Selected sites have had no further harvesting operations over the last decades, and include large tracts of untouched old-growth forests^44^ intermixed with partially harvested forests^46^. In most forests of the region these partial harvests were practiced widely, but not with a specific silvicultural system in mind (i.e., no silvicultural methods were known at that time in the region). Instead, the practice was to cut the best trees for lumber production^43^. As mentioned, these cuts represent a low severity overstory disturbance, in which some of the larger high-quality trees in terms of timber value were removed. On our sites, removal ranged from 4 to 22 m^2^ ha^−1^ of the basal area logged, i.e., around 30% of the total basal area in these forests that can reach values close to 70 m^2^ ha^−1^ (Table 1). Thus, in many aspects these partial harvests likely had similar impacts as harvests operations applied in formal silvicultural systems^1^, e.g., in single tree or group selection cuttings^1^. Since *N. pumilio* and *N. dombeyi* naturally establish well in canopy gaps^47,48^, partial harvesting allowed the development of dense and vigorous tree regeneration especially in moist and humid forests^46^. Many harvested forests now appear to have more irregular structures and visually reflect typical multi-aged stands (Daniel Soto, personal observation). In this regard, they appear similar to old-growth lenga forests in mesic and humid (i.e., ~ 600–1000 mm of MAP) conditions in the region, which have irregular and multi-aged structures^46^. This study^46^ also showed that net primary productivity (based on NDVI as a proxy) was higher at the humid site (~ 1000 mm of MAP) and lower at the dry site (~ 600 mm MAP), therefore suggesting a correlation between rainfall, structure and net primary productivity. Also, for lenga-dominated forests have been described a negative relationship between elevation with precipitation^49^, which is also the case of our study (r = − 0.530, *p* = 0.0162). To explore this further, the present study covered much of deciduous forest of the western Patagonia, where topography, soil, and climate conditions represent typical mountain or cordillera conditions. On the other hand, most of the soil sites correspond to Andisols with sandy loam textures developed over glacial material and classified in the family of frigid humic dystrudepts, and in the mesic and humid sites the soils have sandy textures and finer textures in the upper layers of the soil profile^50^. Some soil measured features and some specific details about the stand variables of the selected plots are given in Table 1. While these were established forests, all sites may experience occasional herbivory by livestock. Further details about the stand dynamics of these forests can be found in Fajardo and de Graaf^47^, Soto et al.^46^, Veblen et al.^48^.

**Table 1:** Sites and forest characteristics for the 20 forest sites studied.

Site	Dominant species	MAP	MAT	Unharvested											Harvested												
				masl	s/a	N	C	FC	Dg	BA	pBA	SSCI	EVI	TR	masl	s/a	N	C	FC	Dg	BA	pBA	Bas	SSCI	EVI	HY	TR
PN Cerro Castillo ‑ Ibañez	Lenga	318,3	5,91	809	17 s	14,7	2,7	18,6	26,4	29,2 (7,6)	100	3,2 (0,25)	0,38 (0,04)	1	763	18 s	7,0	1,4	19,2	60,1	25,6 (13,7)	100	10,0 (9,8)	3,28 (0,45)	0,442 (0,04)	1990	1
Rodeo Los Palos	Lenga	417,6	6,06	1009	4,8 s	20,3	3,9	24,1	51,3	49,2 (5,4)	100	2,90 (0,07)	0,43 (0,07)	1	985	10,5 ne	3,5	9,8	29,2	58,8	31,6 (11,6)	100	13,6 (5,5)	2,8 (0,21)	0,39 (0,03)	1990	1
Coyhaique alto 3	Lenga	481,1	5,47	1040	16,5 e	21,7	4,7	26,9	49,7	36,4 (10,1)	100	3,27 (0,16)	0,38 (0,03)	1	1082	14,7 se	16,8	6,8	30,3	96,3	34,8 (14,4)	100	22,4 (7,1)	4,11 (0,31)	0,41 (0,03)	1990	1
Coyhaique alto 1	Lenga	514,7	5,25	1138	15,7 se	15,4	2,2	26,4	31,3	50,0 (8,6)	100	4,79 (0,41)	0,48 (0,04)	1	1136	15,7 se	16,1	4	24,8	75,8	35,6 (12,8)	100	15,6 (5,9)	2,89 (0,06)	0,42 (0,03)	1992	1
Coyhaique alto 2	Lenga	585,9	5,00	1130	21,2 se	21,7	3	28,5	40,9	48,0 (14,5)	100	3,54 (0,08)	0,46 (0,03)	1	1151	10,6 sw	32,2	8,7	25,3	34,3	55,2 (7,8)	100	16,4 (10,9)	3,24 (0,17)	0,48 (0,04)	1967	1
PN Cerro Castillo ‑ Paso Las Mulas	Lenga	714,8	2,74	1053	6,4 ne	8,4	3	24,6	47,7	53,6 (16,2)	100	3,52 (0,18)	0,53 (0,06)	1	1071	7,3 nw	6,4	9,1	32,3	38,4	56,0 (8,6)	100	14,4 (4,6)	4,95 (0,18)	0,55 (0,06)	1956	1
Tío Lele	Lenga	737,3	5,61	1195	11,6 sw	49,7	7,2	23,4	54,6	71,2 (5,4)	100	3,04 (0,10)	0,49 (0,04)	1	1052	19,3 sw	14,7	5.9	22,6	35,9	55,2 (11,2)	100	6,8 (1,8)	4,47 (0,32)	0,48 (0,03)	1989	1
RN Trapananda ‑ Laguna Rivera	Lenga	756,2	3,28	1083	13,1 w	9,1	3,7	22,7	32,2	50,0 (7,1)	100	3,50 (0,26)	0,45 (0,04)	1	1081	3,5 sw	7	3,8	26,1	28,5	44,8 (4,2)	100	7,2 (1,8)	3,28 (0,13)	0,49 (0,05)	1965	1
PN Cerro Castillo ‑ Laguna Chiguay	Lenga	768,5	3,66	988	11,2 se	16,8	6,4	26,8	20,6	55,5 (5,8)	100	3,36 (0,19)	0,47 (0,05)	1	988	11,2 se	16,8	6,4	26,8	15,3	38,0 (12,6)	100	18,0 (2,8)	3,93 (0,27)	0,58 (0,06)	1965	1
RN Trapananda ‑ Laguna Escondida	Lenga	834,5	3,78	1066	5,1 n	12,6	8,7	25,7	43,7	46,0 (9,6)	100	3,37 (0,35)	0,46 (0,05)	1	994	15,8 n	12,6	8,7	26,7	11,1	37,6 (12,8)	100	19,2 (5,0)	5,30 (0,29)	0,46 (0,05)	1962	1
La Pradera ‑ Mininco	Lenga	854,4	4,71	749	8,5 n	92,4	7,2	24,3	40,2	52,0 (12,6)	100	3,99 (0,16)	0,51 (0,05)	1	752	3,7 s	92,4	7,2	28,3	40,6	50,0 (5,8)	100	8,0 (4,9)	4,03 (0,40)	0,51 (0,05)	1992	1
El Fraile	Lenga	917,2	3,61	1062	8,5 n	20,3	4,6	27,9	22,7	58,5 (4,7)	100	3,42 (0,23)	0,47 (0,06)	1	1005	20,3 se	21,7	5,5	27,4	50,6	52,8 (4,2)	100	11,2 (5,4)	5,37 (0,20)	0,52 (0,05)	1955	1
RN Coyhaique	Lenga	1042,6	5,47	966	12,1 s	16,1	5,7	25,4	60,8	68,0 (12,5)	100	4,50 (0,92)	0,50 (0,04)	1	897	6,8 sw	17,5	5,9	18,1	29,3	73,6 (3,9)	100	4,4 (2,6)	4,67 (0,41)	0,45 (0,03)	1971	1
RN Coyhaique	Lenga Coihue	1109,6	5,47	764	17,9 s	45,4	9,2	23,1	30,4 ‑ 50,7	52,8 (15,3)	60	4,62 (0,31)	0,52 (0,05)	2	708	10,2 s	51,1	6,5	17,7	20,9 ‑ 78,8	58,8 (11,9)	45,3	10,8 (6,9)	5,26 (0,82)	0,58 (0,03)	1967	2
Lago Verde ‑ Mañihuales	Lenga Coihue	1336,5	4,76	758	13,5 nw	26,6	7,4	34,2	44,7 ‑ 53,5	41,6 (8,3)	65	4,79 (0,41)	0,40 (0,03)	2	708	17,4 nw	35	7,1	37,7	39.1–61,7	35,2 (12,5)	55,1	14,8 (5,4)	5,43 (0,25)	0,48 (0,02)	1965	1
Cerro Rosado ‑ San Francisco	Lenga	1423,4	3,99	1172	27,1 nw	33,6	7,1	52,2	40,8	65,6 (11,8)	100	3,22 (0,19)	0,51 (0,06)	1	1059	6,8 s	21,7	5,5	28,3	43,2	49,6 (14,9)	100	7,2 (7,0)	4,43 (0,25)	0,53 (0,07)	1990	1
RN Mañihuales	Lenga	1582,1	3,97	1002	24,9 se	28	6,8	38,8	42,9	54,8 (19,7)	100	4,45 (0,18)	0,46 (0,03)	1	952	14,9 se	31,6	6,3	39,1	42,9	42,4 (9,4)	100	12,5 (5,8)	6,27 (0,48)	0,56 (0,03)	1985	1
San Sebastian	Lenga Coihue	1688,3	4,78	903	20,1 nw	7	5,5	28,7	30,6–55,7	52 (11,9)	47,6	5,53 (0,60)	0,43 (0,01)	2	613	10,4 e	8,4	2,9	24,1	5,65–20,4	34,0 (4,2)	49,1	15,2 (2,3)	8,54 (0,58)	0,55 (0,02)	1992	2
Lago Cofre	Lenga ‑ Coihue	2120,4	5,84	649	11,2 n	12,6	3,4	26,4	34,7–60,3	47,2 (9,4)	51,5	6,63 (0,72)	0,39 (0,01)	3	642	11,7 e	5,7	9,1	32	24,2–61,8	33,6 (4,3)	42,8	14,0 (6,3)	9,16 (1,80)	0,56 (0,03)	1992	4
Valle Laguna	Lenga Coihue	2508,2	5,75	719	9,2 ne	77	30,6	62,9	15,6–52,7	36,8 (7,0)	20,7	6,63 (0,51)	0,46 (0,01)	2	741	9,5 n	11,4	49,7	49,8	8,69–67,2	32,8 (12,8)	22,2	10,0 (4,0)	8,31 (0,62)	0,60 (0,05)	1992	2

*MAP* mean annual precipitation (mm), *MAT* mean annual temperature (°C), *masl* meters above sea level, *s/a* slope in degrees (°)/aspect, *N* total soil mineral nitrogen (mg/kg), *C* soil organic carbon (%), *FC* soil field capacity (%, 330 hPa), *Dg* quadratic stem diameter (cm) for lenga and coihue, *BA* total basal area (m^2^ ha^−1^ ± (SD)), *pBA* proportion of the basal area dominated by lenga (%), *SSCI* stand structural complexity index (mean ± (SD)), *EVI* enhanced vegetation index (mean ± (SD)), *HY* year of the harvesting, *TR* tree species richness, *Bas* mean basal area of stumps (m^2^ ha^−1^ ± (SD)). MAP and MAT obtained from CR2MET raster for the period 1979–2019.

N and C were obtained from a disturbed mix-soil samples located at the 5-scan points in the 1-ha plot. N was determined by Kjeldahl digestion and organic C through wet digestion using Walkley and Black method^51^. Undisturbed soil samples were taken at the centre of each 1-ha plot with a cylinder of 200 cm^3^. In these samples, field capacity (FC: θ_−33 kPa_) and permanent wilting point (PWP: θ_− 15430 kPa_) was determined in laboratory using pressure chambers and pressure plates, respectively^52^. Θ: Volumetric water content (cm^3^ cm^−3^) at each matric potential (− 33 and − 1543 kPa).

### Sampling design and measurements

In 20 locations a pair of 1-ha square plots were sampled in 2 stands. These plots were installed in close vicinity to each other (maximum 1 km), one in an unharvested and one in a partially harvested stand, the latter harvested before 1992 and with no signs of further human-induced overstory disturbances, such as harvesting. These stands are mostly located in national reserves, national parks, and on a few private properties (Table 1). The presence of stumps within plots was used as an indicator of past harvesting. In contrast, plots or forests without any evidence of past harvesting (i.e., stumps) or recent large natural disturbances (e.g., treefalls with more than 5 to 10 years), were considered unharvested. All plots were laid out randomly within areas reflecting the respective conditions (i.e., flat to gentle slope conditions, < 20%, see details in Table 1). The history of the stands was also confirmed by consultations with administrators or landowners (e.g., years of harvesting, and securing that there were no subsequent treatments). The field measurements were conducted between mid-to-late summer (i.e., December to March 2023). Sampling and the laser scanning were conducted under selected weather conditions to avoid potential data noise for the point-cloud data processing, i.e., during times with no rain and wind events.

To assess the three-dimensional (3D) forest structure in each 1-ha plot, a five-on-a-dice-like scheme of terrestrial laser scans was used (sensu ^17^). This approach uses five scan positions within each 1-ha plot starting with one scan in the plot center, and four scans at 42 m distance from the plot center towards plot corners (see details in^23^). This plot design was chosen as it has been used in earlier studies^17,23^ and thus allows direct comparisons of results. Having multiple scanning perspectives provides for a more reliable estimate of the forest structure. However, we did not have resources for more than five scans per plot, given the large number of plots investigated here. Anyways, with 1-ha plots, more scans would have been inefficient, leading only to minor improvements in accuracy, as they would result in large areas being scanned from several directions (scanner range: 70 m in open conditions). The scans were obtained with a FARO Focus M70 3D laser scanner (Faro Technologies Inc., Lake Mary, USA) mounted on a tripod at breast height (1.3 m). The device scans its surroundings based on near-infrared laser light (905 nm wavelength) up to a distance of 70 m and calculates the distance to the objects based on the phase-shift method up to a sub-cm resolution. A field of view of 310 degrees in vertical and 360 degrees in horizontal direction is scanned based on an angular step-width of 0.035 degrees.

### Precipitation

The precipitation data for the different locations under study was obtained from CR2MET (www.cr2.cl/datos-productos-grillados) of the Center for Climate and Resilience Research (www.cr2.cl). The CR2MET consists of local climate data of precipitation and mean and extreme temperatures for a rectangular grid of 0.05° latitude and longitude (i.e., 5 km) for continental Chile from 1979 to 2019. The CR2MET’ precipitation raster uses a statistical regionalization of the ERA-Interim (available data grid of ~ 70 km) which is downscaled using statistical models to transfer the large-scale data into a regional scale of precipitation and is corrected and updated using local standardized weather stations along continental Chile.

### Net primary productivity from enhanced vegetation index (EVI)

To estimate the net primary productivity, the enhanced vegetation index (EVI) was used as a proxy. Vegetation indices are near-linearly related to photosynthetically active radiation absorbed by plant canopies, and they were shown to correlate with net primary productivity^53,54^ and other light-dependent physiological processes occurring in the upper canopy, such as photosynthesis. Vegetation indices can also be integrated with time series analyses to reflect the status of net primary productivity. EVI was recommended over other vegetation indices, such as NDVI due to the poorer performance of the latter in the face of atmospheric noise, saturation, soil background, and other aspects, as discussed in Huang et al.^55^.

EVI varies between − 1 and 1, where higher values represent a higher upper canopy net primary productivity. EVI uses the red and near-infrared band (similar to NDVI), and also uses the blue band as a correction factor, which explains why the range of EVI values is lower than indices such as NDVI, but at the same time it is more precise^55,56^. Data to calculate EVI was derived from Sentinel 2 satellite images, which have a spatial resolution of 10 m (pixel), and a temporal frequency of 5 days. Using Google Earth Engine platform through R software (using the “rgee” package), all available images from Sentinel 2 for the temperate-climate growing season were obtained from the center of each plot (60 by 60 m) to avoid the potential influence of the plot border (see plot scheme in^23^). The spatial data was collected from December 1 2022 to March 31 2023. Before calculating EVI, all images containing clouds were removed. In addition, we used the LOESS statistical method, which allows smoothing the spectral data and reducing the influence of residual clouds and aerosols influences, thus ensuring better data quality^57^. Although the Sentinel temporal frequency is 5 days, this was not always achieved in all plots due to the application of cloud filters (between 4 and 26 dates/images per plot). EVI was calculated as follows:

$$EVI=2.5\times \frac{pNIR-pRED}{1+pNIR+(6\times RED-7.5 \times pBLUE}$$

where (ρ) are atmospherically corrected surface reflectance in the near-infrared band (NIR), the red-edge band (RED), and the blue band (BLUE). Coefficient 1 accounts for upper canopy background scattering, and the blue and red bands coefficients, 6 and 7.5, minimize residual aerosol variations^58^. Finally, for each plot, the average EVI of the dates/images of the entire analysis period was calculated (from December 1, 2022 to March 31, 2023).

### Stand structural complexity index (SSCI)

We filtered the point clouds from the terrestrial laser scanning using the standard filters for erroneous measurements provided by the manufacturer’s software FARO SCENE (Faro Technologies Inc., Lake Mary, USA, v.7.1.1.81) and converted them into xyz-files, which basically transform the spherical coordinates provided by the scanner into Cartesian coordinates. The xyz-files were imported into Mathematica (Wolfram Research, Champaign, USA) to compute the stand structural complexity (SSCI) index (sensu^16,17^). In short, SSCI was calculated based on the average complexity of the shape of vertical cross-sectional polygons through the 3D point cloud of the forest scenes. Therefore, vertical cross-sections were derived from the 3D point cloud in each angular direction captured by the scanner. They represented vertical “cuts” through the forest scene in one direction, reaching theoretically as far as the scanner could measure (~ 70 m). While the scanner originally captured the surroundings with 0.035 degrees angular step width (resulting in 10,240 directions for the full circle), the data was downsampled to a fourth of the original resolution provided during scanning (2560 directions) for increased computing performance. The resulting 2560 directions of measurement were then used to combine the two cross sections from two opposite horizontal directions into a single vertical cross section, resulting in 2560/2 = 1280 cross sections. The cross sections’ shapes were analyzed for their 2D complexity following the FRAC index from fractal mathematics as introduced by^59^. The mean complexity of all cross sections was taken to the power of the natural logarithm (LN) of stand height to scale the complexity measure (FRAC) by the vertical extent of the forest. The interested reader is referred to^16,17^ for details and illustrations. The extremely high frequency of quantifying the cross-sectional complexity of a forest and the average value per scan ensured a high sampling intensity and a stable and reliable estimate of the complexity at the sample point. To gain representative plot values, we used the average of the five scans per 1-ha plot as a real and harmonized measure of structural complexity, allowing a fair comparison between harvested and unharvested plot pairs in the same precipitation site. The 5-scans-per-hectar approach was used in earlier studies and in all cases yielded meaningful results^17,23,60,61^. We performed visual inspection of every scan to ensure that no problems occurred during scanning, for example due to the tripod slowly sinking into the ground. All of the scans were complete and none had integrity problems. We would like to emphasize that the SSCI approach is a sampling approach that describes the structural complexity of the forest at the sampling point and only the forest volume visible from this sampling point is included in the calculation. Therefore, it is important to conduct more than one scan per hectare to gain a meaningful “average” complexity. Objects closer to the scanner receive greater emphasize than objects at larger distances in this sampling approach. As utilized in previous studies^17,23,60,61^ and confirmed by Perles-Garcia et al. (2021) this fact is part of the basis for calculation of SSCI, i.e., the entangledness and associated impacts on the sampling area are measures that allow SSCI to quantify structural complexity. Hence, the concept of the SSCI interprets differences in the range that is visible from the scanner as one element of complexity (density) and this is inherently accounted for in the average of the FRAC value.

### Statistical analyses

Generalized linear mixed models (GLMM) with Gamma distribution family structure and Log link function were used to fit the relationships between (1) mean SSCI (stand structural complexity index, response variable) and mean annual precipitation (MAP in mm, predictor variable), and (2) mean EVI (enhanced vegetation index, response variable) and mean SSCI (predictor variable). In both cases predictor variables are fixed variables. Additionally, harvesting (binary variable: 1 harvested and 2 unharvested plots) was used as an indicator variable. Since paired plots -one harvested and one unharvested- are nested within the same precipitation location, the location of the two paired stands (harvested and unharvested) was used as a random variable^62^. Previous to fit the models, parametric assumptions were tested. Model fitting was conducted through restricted estimated maximum likelihood (REML). For H1 (MAP effect on SSCI especially with harvest), the evaluation of the model’s predictor structures were: (1) MAP only (without distinction with harvesting or all data together), (2) MAP + indicator variable harvest, and (3) MAP*indicator variables harvest. For H2 (SSCI effect on EVI especially with harvest), the evaluation of the model’s predictor structures were: (1) SSCI only (without distinction with harvesting or all data together), (2) SSCI + indicator variable harvest, and (3) SSCI * indicator variables harvest. Last, for H3, the triple interaction of SSCI + MAP + harvest effect on EVI, we evaluated four different predictor combinations: (1) SSCI + MAP, (2) SSCI*MAP, (3) SCI + MAP + harvest, and (4) SSCI*MAP*harvest. The corrected Akaike information criterion (AICc), delta AICc, log likelihood (LogLik), and root mean square error (RMSE) were used for assessing the best supported models per each hypothesis. Additionally, we obtained the conditional coefficient of determination (cond. R^2^) and marginal coefficient of determination (marg. R^2^), where the cond. R^2^ takes both fixed and random variables into the proportion of variance explained, and the marg. R^2^ just means the proportion of the variance explained by the fixed variables only^63,64^. These approaches allowed us to consider the influence of random variable proposed on overall model fit. The interpretation of these coefficients is equal to classical R^2^. The differences among model structures grouped per hypothesis were evaluated through chi-square tests at 95%. GLMMs were run using R studio platform with packages “lme4” package for fitting the models^65^, “performance” package for the model evaluation^66^, and “effects” package for plotting the best supported models^67^.

## Results

### Relationships between SSCI with MAP and harvesting

**Figure 2:**
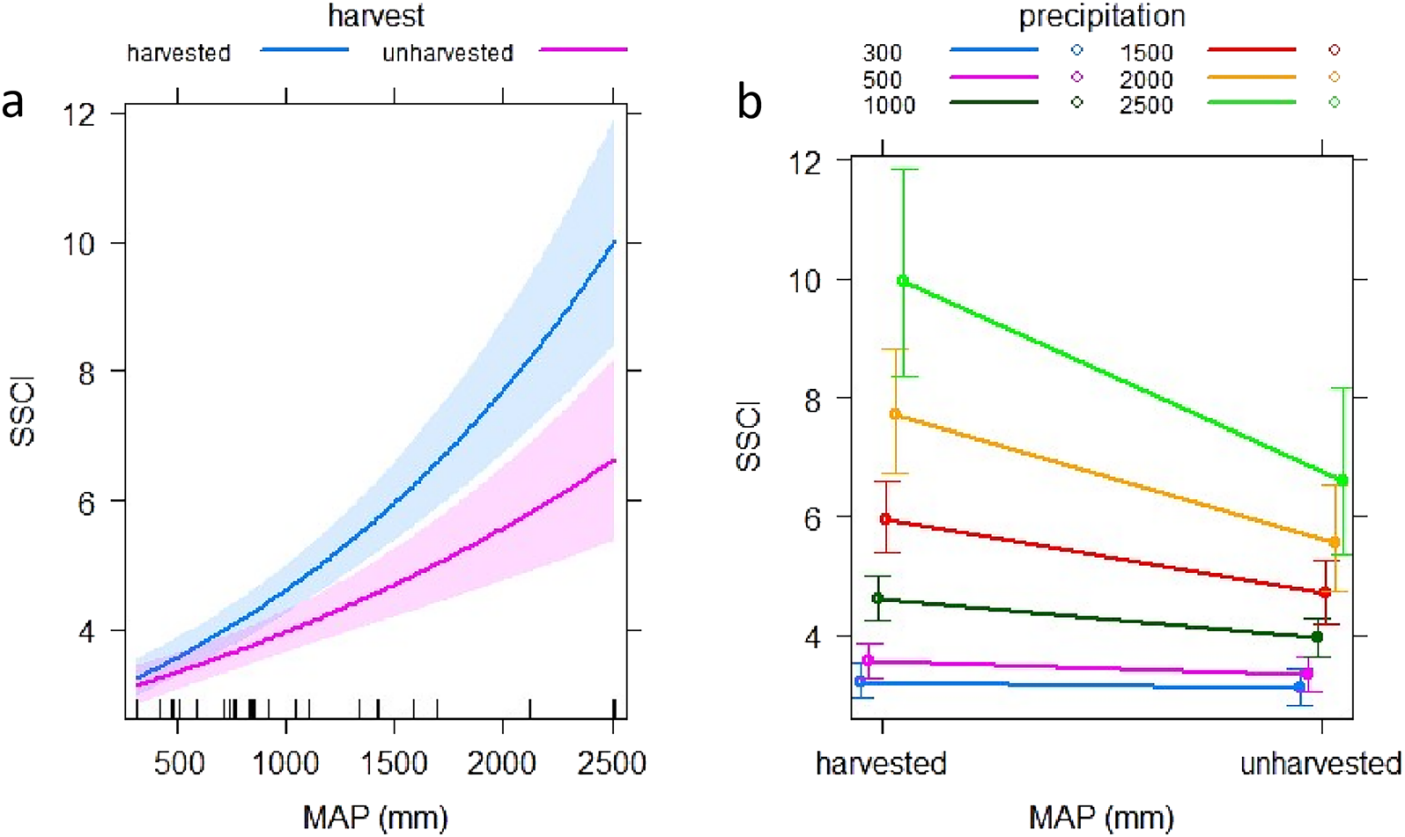
Mean annual precipitation-MAP (mm) and harvest predictor effect plots for the best supported model for the prediction of SSCI. Color bands in (**a**) represent the 95% Cis for harvested (blue) and unharvested (red) plots. (**b**) Shows predicted values at selected MAP levels, whereby the error bars represent the standard deviation.

SSCI increases along the MAP gradient studied, and this relationship is steeper in stands that were partially harvested (Fig. 2). The best supported model of SSCI included the interaction of MAP (multiplication) with the indicator variable harvest (model 3 in Table 2; cond. R^2^ = 0.802). This model was better supported (i.e., delta AICc > 2) than model 1 that used only MAP and model 2 that included MAP + harvest (Table 2). The best supported model had the lowest LogLik, AICc, and RMSE providing strong empirical support (Table 2). The 95% confidence intervals (Cis) between harvested and unharvested plots did not overlap above ~ 1000 mm of MAP (Fig. 2a). To visualize how the strength of the effect of harvesting on SSCI increased with higher MAP, we displayed predicted SSCI (using the best supported model) for selected levels of MAP in Fig. 2b. On the other hand, the marg. R^2^ shows that the inclusion of a fixed variable only in the model has a lower efficiency than including fixed plus random variables in the model, which explained an additional 7.2% of the variance (Table 2). Graphical residual evaluations for the best supported model are given in Supplementary Fig. S1.

**Table 2:** Statistical summary of all models evaluated for SSCI (H1) and EVI (H2).

Models	LogLik	AICc	Delta AICc	Cond. R^2^	Marg. R^2^	RMSE	Chi^2^	*P*-value
1. SSCI ~ MAP	− 47.8	104.9	11.9	0.689	0.641	0.818	–	–
2. SSCI ~ MAP + harvest	− 42.1	95.9	2.9	0.770	0.703	0.624	11.6	0.0006
3. SSCI ~ MAP*harvest	**− 39.3**	**93.0**	**0**	**0.802**	**0.730**	**0.572**	**5.6**	**0.0180**
1. EVI ~ SSCI	73.4	− 137.8	12.1	0.775	0.461	0.028	–	–
2. EVI ~ SSCI + harvest	75.1	− 138.6	11.6	0.741	0.425	0.027	3.4	0.0651
3. EVI ~ SSCI*harvest	**82.2**	**− 149.9**	**0**	**0.747**	**0.417**	**0.023**	**14.1**	**0.0001**

*The best supported models per hypothesis is highlighted in bold.

### Relationship between EVI with SSCI and harvesting

**Figure 3:**
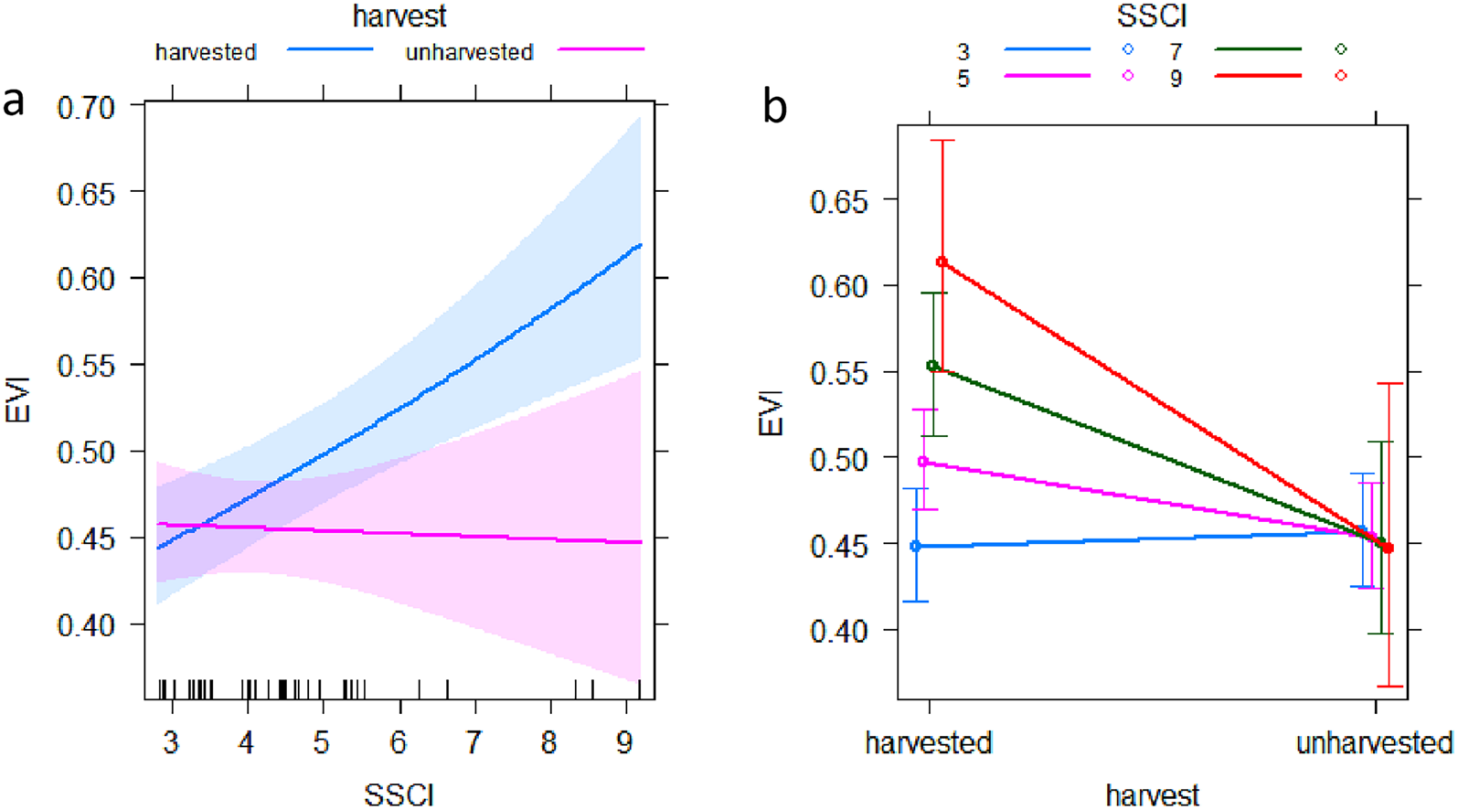
SSCI and harvesting predictor effect plots for the best supported model for the prediction of EVI. Color bands in (**a**) represent the 95% Cis for harvested (blue) and unharvested (red) plots. (**b**) Shows predicted values at selected levels of SSCI, whereby the error bars represent the standard deviation. The error bars in panel b represent the standard deviation.

Our results indicated that higher SSCI is related to higher EVI, but only in stands that were partially harvested (Fig. 3). The best supported model for the prediction of EVI was model 3 (Table 2), which also included the interaction (multiplication) of SSCI with the indicator variable harvest (cond. R^2^ = 0.747). Moreover, Fig. 3a shows that the 95% Cis of harvested plots and unharvested plots did not overlap after SSCI reached values of six or greater. The best supported model 3 had the lowest AICc (all delta AICc > 2) and RMSE, providing strong empirical support compared to alternative models (Table 2). To visualize how the strength of the effects of harvesting on EVI varied with SSCI, we displayed predicted EVI values for selected levels of SSCI (using the best supported model) in Fig. 3b. The strength of the effect of harvesting resulted in higher EVI with higher SSCI, but it remains almost invariant along SSCI gradient for unharvested plots (Fig. 3b). On the other hand, the marg. R^2^ shows that the inclusion of a fixed variable only in the model has a lower efficiency than mixed (i.e. fixed plus random variables) model, which explained an additional 33% of the variance (Table 2). Graphical residual evaluations for the best supported model are given in Supplementary Fig. S1.

### Relationship between net primary productivity, stand structural complexity, precipitation and harvesting

**Figure 4:**
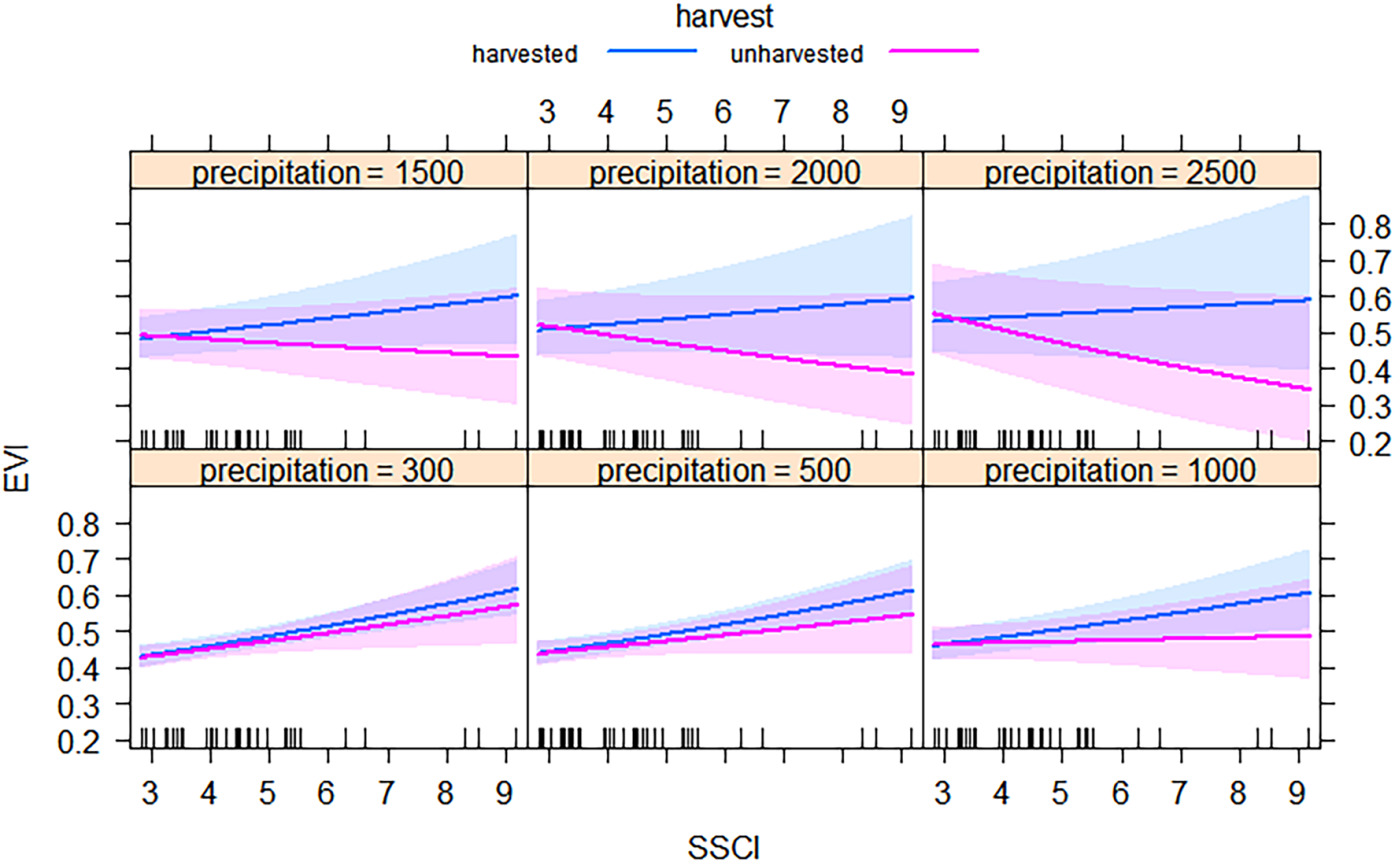
Predicted EVI (using the best supported model) for unharvested and harvested stands and selected precipitation levels over a gradient of stand structural complexity index (SSCI). Color bands represent the 95% CIs for harvested (blue) and unharvested (red) stands.

Model 4 (Table 3) which included the triple interaction between SSCI*precipitation*harvest (indicator variable) was best supported by the data (lowest AICc, delta AICc > 2, and higher cond. R^2^ = 0.806). On the other hand, for this model the marg. R^2^ shows that the inclusion of fixed variable only had a lower efficiency than the mixed (i.e. fixed plus random variables, cond. R^2^) model, which explained an additional 41.3% more variance (Table 3). Figure 4 displays how the triple interaction as it relates to EVI along the SSCI gradient using six selected MAP classes (Fig. 4). This figure shows that EVI from dry (~ 300 mm) to mesic conditions (~ 1000 mm) for harvested and unharvested plots had a neutral or positive relationship with SSCI, being a little more positive for the harvested plots (Fig. 4).

**Table 3:** Statistical summary of all models evaluated to predict the net primary productivity (EVI).

models	LogLik	AICc	delta AICc	cond. R^2^	marg. R^2^	RMSE	Chi^2^	*P*-value
1. EVI ~ MAP + harvest	− 42.1	− 139.5	8.3	0.711	0.341	0.027	–	–
2. EVI ~ MAP*harvest	− 39.3	− 144.6	3.2	0.752	0.477	0.023	7.06	0.007
3. EVI ~ MAP + SSCI + harvest	75.1	− 139.7	8.1	0.706	0.347	0.027	0.00	–
4. EVI ~ MAP*SSCI*harvest	**82.2**	**− 147.8**	**0**	**0.806**	**0.393**	**0.022**	**16.1**	**0.002**

*The best supported model is highlighted in bold.

The EVI values were similar on harvested and unharvested plots in drier conditions. However, the trends became negative on humid sites, whereby the EVI values on harvested sites were substantially larger than on unharvested sites. Also, the EVI in unharvested stands was consistently negatively related to SSCI on humid sites, i.e., above 1500 mm MAP (Fig. 4). Despite this, we have no statistical support showing that the EVI of harvested plots are different from unharvested ones since the confidence intervals (CIs 95%) overlapped throughout the SSCI and MAP gradients (Fig. 4). Last, graphical residual evaluation for the best supported model is given in Supplementary Fig. S1.

## Discussion

Managing forests to increase forest structural complexity is becoming a more common goal worldwide^5,15,68^. Such silviculture aims at increasing the resiliency and adaptability of forest following disturbances, while maintaining or increasing forest functions^3,6,7,15,18,69^. The relationship between structural complexity on forest productivity has also been documented^29,32^. Recently, Ehbrecht et al.^17^ suggested that precipitation was the major driver of forest structural complexity in primary forests at a global scale. However, long-term influences of harvesting on forest structure and the associated relationship to net primary productivity remain largely unexplored (but see^16,31,35^). The present study addressed this knowledge gap by using old-growth forest stands in the temperate region of western Patagonia that were partially harvested more than 30 years ago, i.e., some decades have passed to evaluate how mid- to long-term changes, such as forest structure, have developed.

Our results that showed increases in SSCI with higher MAP at a regional scale support the findings of the global assessment conducted by Ehbrecht et al.^17^. Since both studies used a similar precipitation gradient but in areas with different other environmental factors, such as temperature, elevation, and soils, we hypothesize that the amount of precipitation was most influential in driving this relationship. Similar patterns were documented for understory complexity along precipitation gradients in central to south-central Chile^23^. It is further reassuring in the present study that findings from both treatments, i.e., in harvested and unharvested stands, support this hypothesis. While there was an increasing SSCI from dry to humid sites, harvesting only significantly increased this difference between treatments in mesic and humid sites, i.e., above 1000 mm of MAP. The consistency of these results suggest that we can downscale the findings from a global^17^ to a local scale^23^ in stands with various small scale disturbance histories.

**Figure 5:**
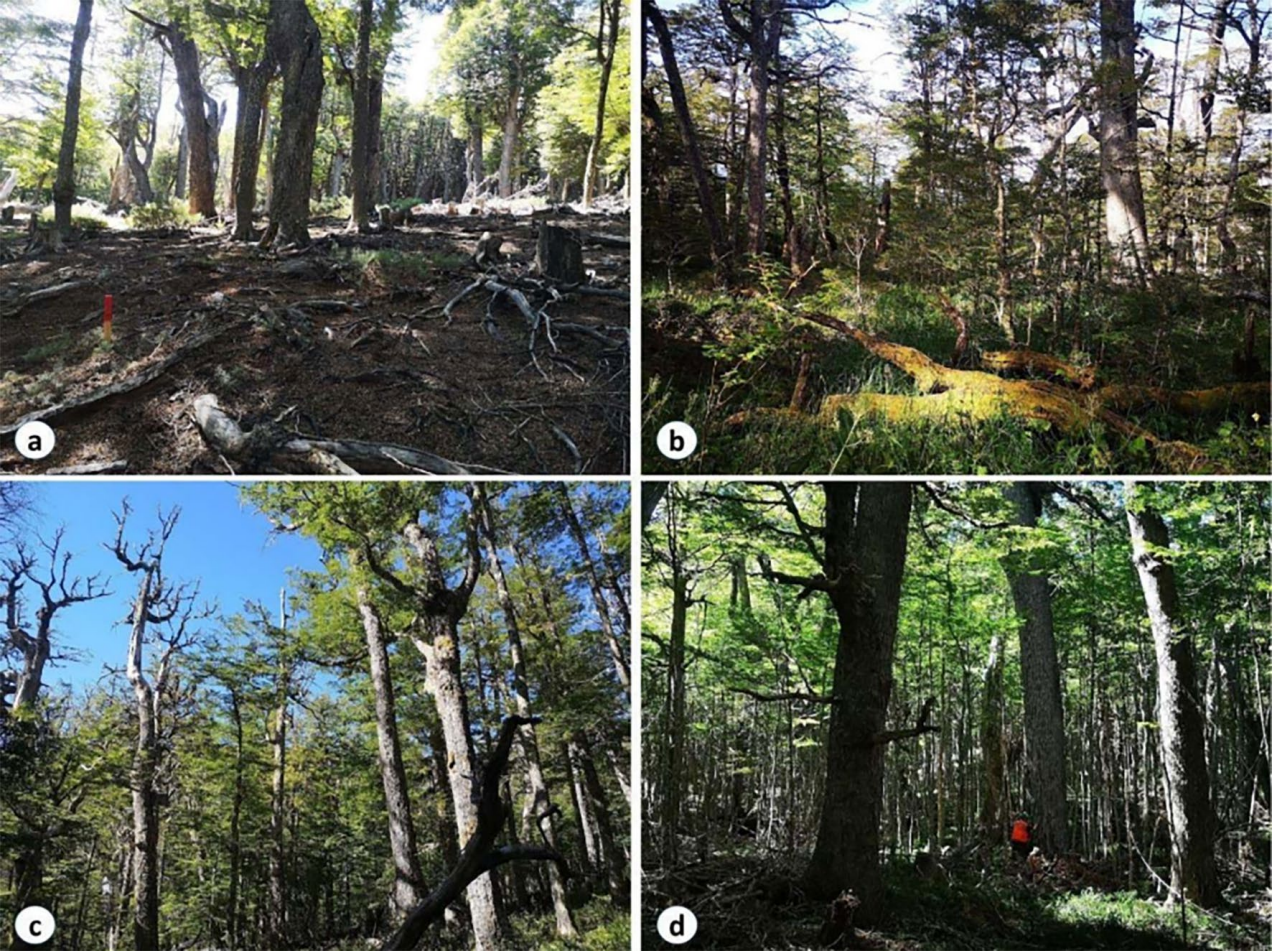
Pictures showing different stand conditions. (**a**) Lack of regeneration and poor stand structural complexity in a harvested plot at a dry site (~ 500 mm of MAP), (**b**) high-structural complexity but low productivity in a unharvested humid site (~ 2500 mm of MAP), (**c**) recent large-tree mortality in a mesic (~ 1300 mm of MAP) unharvested forest which may have affected net primary productivity, and d) dense tree regeneration in a harvested mesic site (~ 1000 mm of MAP) that increased stand structural complexity and net primary productivity.

The tree species that dominated our study sites are known to regenerate better in canopy gaps (sensu^47,48^). Thus, our findings suggest that canopy gaps created following the partial harvest in many cases matched regeneration requirements of these species, and time (> 30 years) allowed new cohorts (or some advanced but stagnated regeneration) to develop and increase stand structure complexity. On the more humid sites, the higher likelihood of a species mixture (coihue, in addition to lenga) and the ability of more vigorous regeneration to recover from herbivory^70^ also likely strengthened these trends. Moreover, a successful regeneration in even-aged stands managed using shelterwood and seed tree silvicultural systems has been well-documented in lenga-dominated forests in Chile and Argentina^71,72^. Also, single-tree selection cuts have been implemented in the Argentinean^73^ and Chilean Patagonia (Daniel Soto, personal observation), with successful regeneration in more humid sites. In contrast, partial harvesting treatments associated to the present study were more ad-hoc, i.e., not designed with silvicultural methods for tree regeneration. Instead, these partial harvests likely resulted in a more variable/heterogeneous set of forest conditions, including canopy gaps of different sizes and shapes, and untouched areas. On mesic and humid sites, but not on the drier sites, gap creation was apparently suitable for tree regeneration, which in the long-term developed and contributed to the creation of more complex forest structures (Figs. 1, 5d). The resulting “dense canopy packing” fills more canopy layers along the vertical axis, contributing to a higher stand structural complexity than found in closed-canopy stands without a history of harvesting^15,17^. The role of regeneration in determining development of forest structures was also found in mesic (~ 800 mm of MAP) and humid (~ 1000 mm of MAP) uneven-aged forests, but not on the dry site (~ 600 mm of MAP)^46^. In the dry site, the regeneration was basically absent, leaving gaps after tree fellings that were not filled with tree regeneration. The lack of regeneration under undisturbed overstories and after partial overstory disturbances on dry sites may have been partially driven by the inability of seedlings in highly competitive or stressful conditions to recover from herbivory and appears to be key for the development of forest structures with low structural complexity. Just as with the global analysis, such findings illustrate the relationship between precipitation (or water availability) and forest structural complexity. Our findings suggest the hypotheses that this is due to the influence of rainfall on the regeneration process (through species composition and seedling vigor) and subsequent vegetation development after partial disturbances.

A contrasting effect of SSCI on EVI in harvested and unharvested plots was evaluated. A good fit was obtained for the relationship between EVI (response variable) with SSCI (predictor variable) using harvest type as indicator variable, accounting for 74.7% of the total variance explained (Table 2). This pattern supports our hypothesis about the relationship between precipitation amount and productivity being influenced by past disturbances (H2). In contrast to the harvested stands, the pattern for unharvested stands had a flat curve without a clear relationship between rainfall and SSCI (Fig. 2) as well as between SSCI and EVI. This pattern is contrary to findings from earlier studies that observed a positive relationship between structural complexity and forest productivity for sub-continental temperate forests ecosystems of North America^32^, as well as for tropical forests in Brazil^34^. In fact, a theoretical framework as to why greater complexity results in greater productivity is emerging (see for example^15^), but more experimental work is need to fully investigate the mechanisms. Locally, Soto et al.^46^ evaluated the net primary productivity through the NDVI in three narrow but distinct precipitation sites (i.e., ~ 600, ~ 800, and ~ 1000 mm of MAP), where sites with higher precipitation had higher net primary productivity and narrow data dispersion throughout the growing season, while sites with lower net primary productivity were located at drier conditions with a higher data dispersion, showing a great variability of the net primary productivity throughout the growing season and specific site conditions. This is potentially an effect of canopy gaps that are unfilled with regeneration after small-scale overstory disturbances, species composition, lower tree densities^46^ possibly due to dieback of large trees due to extreme weather events^74^ (e.g., heat waves or drought events during the growing seasons), or combinations thereof (Fig. 5). Clearly, empirical studies are needed to investigate the how the relative role of these factors plays out in various settings.

Interestingly, harvested plots showed a positive relationship of stand structural complexity with net primary productivity, with important implications to the adaptability of these forests in the long-term (see Fig. 5d). This suggests that the new cohort of vigorous tree regeneration was successfully established in mesic and humid sites, and likely these portions of the stand with younger trees enhanced the net primary productivity of the stands^75^ and also contributed to a higher stand structural complexity at the plot scale^15,22,29,32^. More detailed data on growth patterns of the different canopy layers are needed to confirm this. Therefore, in regards to stand dynamics, one can view the unharvested stands (i.e., in absence of any kind of disturbances, small to large scale) as being in relatively stable late successional conditions, described as being in a “rigidity trap” with high connectedness (sensu^69^). In this context, partial harvests are disturbances sufficiently severe to lower connectedness and allow tree regeneration and successional development, which results in higher stand structural complexity. The regeneration in canopy gaps could also be operating as a driver to enhance net primary productivity at mesic and humid sites (see Fig. 5 for more pictorial details). In contrast, the vegetation development in unharvested stands which apparently did not overcome the rigidity trap may also be reflected in lower productivity^69^.

The best supported model of net primary productivity included a triple interaction between SSCI, MAP and harvesting (cond. R^2^ = 80.6%). We saw a generally decreasing trend of EVI as a function of increasing SSCI and MAP for unharvested stands, but not for harvested ones. However, these differences were not statistically significant (Cis overlapped throughout the SSCI gradient) (Fig. 4). Therefore, the statistical evidence does not support hypothesis 3. A similar negative trend between net primary productivity and precipitation was found along an ample precipitation gradient (i.e., 2000–5000 mm of MAP) in Hawaiian montane wet forests^76^. There, an increased water availability that exceeds plant demand can produce a detrimental effect on net primary productivity by, e.g., soil's redox potential, which affects soil decomposition, oxygen regime and nutrient availability (e.g., soil and leaves N) with higher precipitation.

It is well known that climate drives the net primary productivity globally^77,78^. Specifically, precipitation is positively related to net primary productivity up to 2000 mm of MAP, and the relationship abruptly switches to a negative relationship beyond this threshold^78,79^. Therefore, our study confirms that below 1000 mm of MAP in forests with and without a harvesting disturbance, these are likely water-limited forest ecosystems in terms of tree productivity^77^. Our findings also suggest the hypothesis that with higher precipitation (e.g. above 1500 mm of MAP), forest structural complexity development and net primary productivity become more nutrient-limited forest ecosystems^77^. However, in this regard, the influence of harvesting and its subsequent influence on vegetation development may override this trend. Our findings suggest that an investigation into the water and nutrient status of the trees may provide more insights, especially in stands with greater forest complexity and higher precipitation.

### Implications for forest management

The increases of stand structural complexity and net primary productivity when MAP is medium to high in stands after partial harvestings provide important insights to the design of silvicultural treatments. These results illustrate the potential opportunities for and benefits of multi-aged silviculture in forest stands. Stands managed with multi-aged silviculture can provide more selected ecosystem services compared to even-aged stands^1,7^ and there is an increasing scientific agreement that these forest structures are more adaptive and have a greater role in mitigating climate change^4,11^. This difference is partially explained by the structure of multi-aged stands and our findings illustrate that the complexity of this structure can be boosted through partial harvestings. In particular, single-tree and group selection methods could be an option in mesic and humid sites throughout the region of *Nothofagus pumilio*-dominated mature and old-growth forests. In addition, shelterwood methods without a final cut (such is irregular shelterwood cuts) seem promising since their application in southern South America has shown a great success in terms of tree regeneration and recovery trends^71,80^. Both systems are in line with recent advances in ecological silviculture (sensu^4^). The fact that more recent partial overstory disturbances encouraged greater structural complexity and were related to productivity compared to unharvested older stands in areas with medium to high precipitation illustrates the potential benefits of active and continuous silviculture, e.g., through the implementation of successive entries or cutting cycles within a selection system.

Despite these results, special attention should be paid to drier forest conditions with poor structural complexity that was not enhanced with partial harvesting. These systems have little resiliency and are highly vulnerable to global change stressors^46^, e.g., by providing for more continuity in fuels^81^, higher likelihood of damage in windstorms^82^ and limiting the potential recovery or developmental pathways^83^. Thus, it may be more important than on more humid sites to reduce or eliminate additional stressors after harvesting, such as herbivory by livestock. In these dry forests, developmental patterns are similar to those found in forests with a mixed severity disturbance regime, i.e., extended recruitment periods follow partial overstory disturbances which eventually can generate complex structures^83^. Thus, our finding provides insights on how forest management can influence future development of stand structures and productivity in the context of the predicted climate change in western Patagonia, i.e., lower precipitation and increasing temperature^74^. Specifically, forest management in Patagonia should avoid additional stressors and promote resistance and resilience adaptation strategies to trigger regeneration as a basis to develop complex structures and productive forest stands^3–6^.

Finally, since we used harvest as an indicator variable, but did not analyze structural patterns along the harvested plots that spanned from 1955 to 1992, we cannot comment the magnitude of a time-since-harvest effect. However, at least for moderate-severity partial harvests, and considering the relatively low growth rates of Patagonian forests, our study appears to show an example how these cuttings broke the rigidity trap (see text above) that occur with regeneration dynamics in old-growth forests. Addressing this issue on a landscape scale poses a significant study challenge, since it is likely that periodic (or continuous) artificial disturbances, such as partial harvests with scientific bases (i.e. silvicultural approaches) might be needed. Moreover, despite all efforts to limit our work to lenga monocultures, we had to compromise and include five sites with mesic and humid conditions (above 1000 mm of MAP) that were occupied by mixed lenga-coihue stands. This raises questions about the potential role of tree diversity on stand structural complexity (see^33^) as related to climate gradients (e.g., precipitation). For example, one approach could be to expand the future sampling efforts to species mixtures, ideally of these two species, in western Patagonia with a wider precipitation gradient, i.e., up to and above 4000 mm of MAP. As with all studies using extensive field sampling, a cautionary note for this study is related to the methodological constraints. These include the number of sampled stands, the low number of scans (n = 5) per 1-ha plot, the use of a proxy of productivity instead field measurements of net primary productivity, and the use data from meteorological stations instead raster data. Future research is needed to investigate the implications of such limitations. However, acknowledging potential limitations and the new questions this study raised, it provides important insights to refine theory and to sustain some silvicultural alternatives to enhance forest structural complexity and net primary productivity along a precipitation gradient in the western Patagonian forests.

### Supplementary Information

Supplementary Figures.

## Data Availability

Data availability upon request to the corresponding author.
