# Supplementary material for: Increase in forest structural complexity along a precipitation gradient is mediated by partial harvests in temperate Patagonian forests
Source: Sci Rep. 2024 Jun 13;14:13656. doi: 10.1038/s41598-024-64523-5 (PMC11176304; doi:10.1038/s41598-024-64523-5)
Supplement: Supplementary file 1 — Supplementary Figures. [file 41598_2024_64523_MOESM1_ESM.pdf]

Increase in forest structural complexity along a precipitation gradient is mediated by partial harvests in temperate Patagonian forests

Daniel P. Soto[1],*, Dominik Seidel[2], Ángela Hernández-Moreno[3], Klaus J. Puettmann[4], Pablo J. Donoso[5]

[1] Departamento de Ciencias Naturales y Tecnología, Universidad de Aysén, Coyhaique, Chile
[2] Department for Spatial Structures and Digitalization of Forests, Georg-August-Universität, Göttingen, Germany
[3] Centro de Investigación en Ecosistemas de la Patagonia (CIEP), Coyhaique, Chile
[4] Department of Forest Ecosystems and Society, Oregon State University, Corvallis, Oregon, USA
[5] Instituto de Bosques y Sociedad, Universidad Austral de Chile, Valdivia, Chile
* corresponding author: daniel.soto@uaysen.cl

Supplementary materials

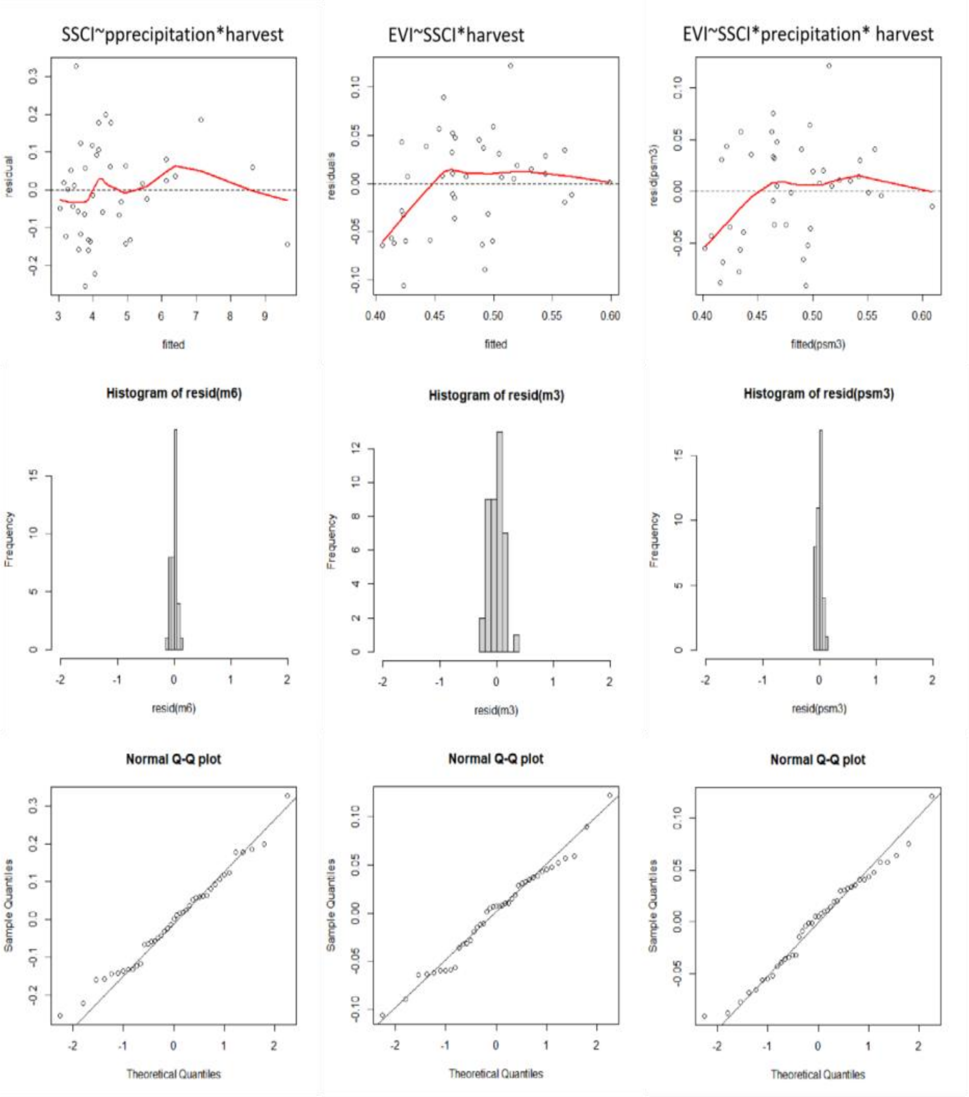

Fig. S1. Residual evaluation for the best supported models for each hypothesis. H1: SSCI (stand structural complexity index) is positively related to precipitation and this relationship is influenced by stand history (left panels). H2: Net primary productivity (i.e., EVI) is positively related to SSCIentral panels). H3: A positive triple interaction between SSCI, precipitation and partial harvesting influences the net primary productivity (EVI) (right panels).

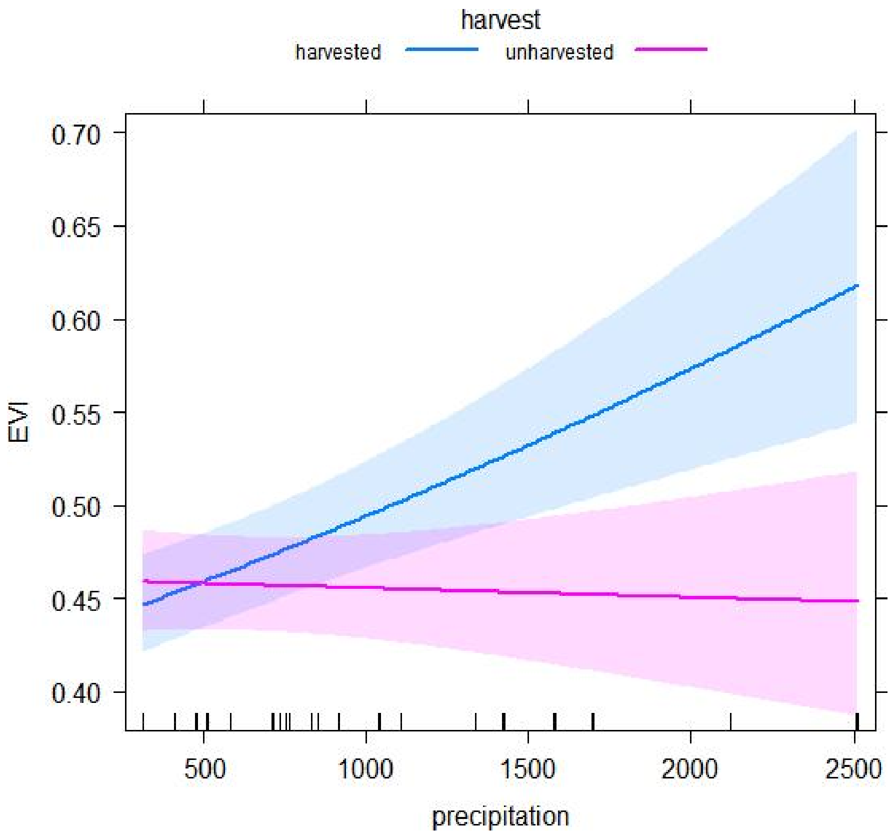

Fig. S2. Precipitation (mm) and harvesting predictor effect plot for the fit of EVI. Color bands represent the 95 % CIs for harvested plot (blue) and unharvested plots (red).
